# An Innovative Approach to Surveying the Geometry of Visibility Triangles at Railway Level Crossings

**DOI:** 10.3390/s20226623

**Published:** 2020-11-19

**Authors:** Arkadiusz Kampczyk

**Affiliations:** Department of Engineering Surveying and Civil Engineering, Faculty of Mining Surveying and Environmental Engineering, AGH University of Science and Technology, al. A. Mickiewicza 30, 30-059 Krakow, Poland; kampczyk@agh.edu.pl

**Keywords:** roads, railroads, intersection angle, triangle of visibility, safety roads, rail safety, accidents and incidents, railway level crossing, safety challenge, control measurements

## Abstract

Railway level crossings (RLCs) in Poland are classified according to their protection systems. Category D, which is a form of passive RLC, aims to ensure safe and efficient operation. Surveying is essential to prepare and control the geometry of the visibility triangles used at RLCs. This article presents a new approach to monitoring the geometry of visibility triangles of RLCs using an electronic total station and a magnetic measuring square (MMS). Its main assumptions are presented together with the application of the innovative measuring instruments. Visibility is demonstrated taking into account the angles of intersection of the road axis with the track axis of the railway line and additional attributes related to the analysis and evaluation of general visibility conditions. The research highlights controversies that have received special attention against the background of the safety status of railway level crossings. As a case study, the RLC located on a single-track railway line in Poland is examined. The final section presents applications of the results obtained according to the proposed methodology. It is shown that the proposed approach is practical and effective. In addition to surveyors, the survey methodology can be used by road and rail traffic engineers and policy makers to further improve traffic safety at RLCs. This is an important global research task.

## 1. Introduction

Railway level crossings (RLCs) are intersections of railway lines with roadways, other than pedestrian crossings (i.e., crossings, cross-walks, or transitions), on one level. A pedestrian crossing comprises a crossroads on one level intended exclusively for pedestrian and/or bicycle traffic [1]. RLCs and pedestrian crossings in Poland are divided into the categories A–F as follows [1,2,3,4]:

1. A—RLC on which the road traffic is directed by an authorized employee;
2. B—RLC on which the road traffic is directed by means of an automatic crossing system (ACS) equipped with traffic signals (roadside signals) and barriers;
3. C—RLC on which the road traffic is directed by means of an ACS equipped only with traffic signals;
4. D—RLC that is not equipped with motion protection systems and devices;
5. E—a pedestrian crossing equipped with:
  - semi-automatic or automatic crossing system;
  - reels, barriers, or mazes;
6. F—RLC or pedestrian crossing located on an internal road, equipped:
  - with barriers, permanently closed, and opened by users as needed;
  - in accordance with the technical conditions specified for category A or B.

The basic classification of railway level crossing protection systems is divided into passive and active [5,6]. In passive systems, the road user is solely responsible for observing the traffic situation (e.g., an approaching train). Road traffic signs used for passive level crossings (i.e., traffic signs “Stop” and “St. Andrews Cross”) are unchanged and users must understand their meaning, look/listen for trains, and then respond according to the observed traffic situation. By comparison, an active protection system changes its state to warn road users of an approaching train (i.e., via flashing lights and sound and/or full or half barriers) [5,6].

RLCs pose a significant safety challenge due to complex social and technical systems that involve interactions between many different types of road users (pedestrians, cyclists, motor vehicle drivers) and rail operators (drivers, signaling operators) and infrastructure (protective systems) [5,7].

Abnormalities at railway level crossings are a global problem. Nedeliaková et al. [8] emphasize that the safety of railway level crossings is a global concern. Because individual railway level crossings have specific conditions, it is necessary to monitor each of them individually. Tjahjono et al. [9] recognize that accidents between road and rail traffic at railway level crossings are an important problem in developing countries. Although accidents are a major problem for railway companies globally, compared to road accidents, the number of accidents at railway level crossings is very small [10]. Ambros et al. [11] and Tey et al. [12] state that railway level crossings are a critical and complex element of both rail and road networks, at which accidents in most cases lead to serious and fatal injuries. The results in [11] confirmed the influence of traffic flows on safety, in addition to speeding and individual viewing distances. Evans et al. [13] referred to the relationship between railway level crossings, delays, and fatalities for road users at railway level crossings in the United Kingdom. The choice between automatic or rail-controlled RLCs on public roads was considered. Liang et al. [14] confirm that safety is a key issue in railway operations. In particular, safety at RLCs is one of the most critical points on railways.

RLCs are critical points with a high number of accidents per year due to the intersection of rail and road infrastructure, and improving their safety is of key importance globally [5,7,15,16,17]. Together with tunnels and specific road black spots, railway level crossings have been identified as a particularly weak point in road infrastructure which seriously threatens road safety [18,19,20,21].

Żakowska [22] states that one of the dominant causes of loss of life and health of people around the world is road traffic. In the group of European Union countries, Poland has for years been a leader in terms of fatal road accidents. The statistics present the importance of the road transport safety problem as an important social problem. The state of transport safety includes two types of safety:

- objective—defined by objective parameters of the traffic environment and the behavior of its participants;
- subjective—assessed in accordance with the feeling and perception of safety perceived by individuals and groups of people.

According to Żakowska [22] and Olejnik et al. [23], an unconscious danger may cause a subjective feeling of safety. With regard to traffic participants, we observe behaviors that they think are safe. In fact, their feeling is subjective, but they do not realize it. Żakowska in [22] and Reason in [24] emphasize that a safe society is an informed society, one that is always aware of where the dangerous edge is, without having to cross it. The edge lies between relative, subjective security and not an acceptable threat. Moving in this area requires special skills and social culture.

Żakowska states in [22] that a change in a driver’s behavior occurs as a result of changes in perceived risk. Subjective perceived risk, depending on the perception processes, is the basis for a driver’s safe behavior. The difficulty of determining the state of subjective security is still controversial. While some scientists claim that drivers in most cases are not aware of the risk they are taking, then exposing other users to danger, other experts express the opinion that normal drivers always make decisions that are not dangerous in their opinion [22,25]. It is also suggested that normal road users accept a certain level of risk, regardless of the existing situation on the road [22,26]. Wilde in [27] states that risk homeostasis as the degree of risk-taking behavior and the magnitude of loss due to accidents and lifestyle-dependent disease is maintained over time, unless there is a change in the target level of risk.

Transport safety issues, especially the theory of driver behavior in the traffic environment, have been referred to in [28], the theory of behavioral adaptation [29], the theory of homeostasis [26,27,30,31], and the theory of risk compensation [32]. In [33], reference is made to how homeostasis drives behavioral adaptation. Ward et al. [34] relate how drivers approach behavior at an unprotected railway crossing before and after enhancement of lateral sight distances as an experimental investigation of a risk perception and behavioral compensation hypothesis. They note that common sense dictates that railway crossings with restricted lateral visibility should be more hazardous than sites with unrestricted visibility. However, evidence of any relationship between lateral sight distance and accident history has not been consistently demonstrated. Based on the tenets of risk homeostasis theory, this relates to disassociation in terms of motorist compensatory behavior (e.g., speed reduction) in response to the perceived risk associated with restricted visibility. The expectation of this regulatory mechanism is to sustain a more-or-less constant safety margin. In this study, we examine the effect of enhancing lateral sight distances at an unprotected crossing. Parallel observations were made at an untreated site to control for secular confounding. Improvement of lateral sight distances resulted in an upstream shift toward longer search durations and a tendency toward faster approach speeds, but it failed to produce a calculated net safety benefit. A survey of local residents suggests that the enhancement treatment reduced perceived risk [34].

In the paper [22,35], it is also noted that a high level of subjective risk, i.e., lack of safety, according to the Klebersberg risk model is often undesirable from the perspective of traffic safety.

Safety in the traffic environment depends on the interaction between an objective threat and a subjective risk. If the impression of risk on the road remains high for an extended period of time, then the driver acts in a state of above-average danger, which can cause objectively dangerous reactions and behavior. Maintaining the driver’s average state of arousal should therefore be considered in the process of designing a safe road environment [22].

Ishak et al. [36] refer to the development of the RLC safety assessment model for evaluating the performance and reliability of RLC locations using stochastic Petri nets. They draw attention to the interconnection of elements in the RLC system, such as signaling control and train and traffic characteristics. The use of a geographic information system (GIS) in the spatial representation of an RLC combines model output with the visualization of the surrounding land use environment and aims at increasing the understanding of RLC accident phenomena.

Pen et al. [37] investigate RLCs by measuring the deflection of railway tracks during a train’s passage using geophones, cameras, and digital image correlation (DIC). Special emphasis is placed on the measurements of track movement during the approach to a railway level crossing before and after tamping.

The impatience and frustration of road users has been identified as a factor underlying non-compliance and can be characterized as a specific risk factor [38,39]. Subjective data showed that participants do not comply with railway level crossing rules due to factors such as time pressure, impatience/frustration, and low perceived risk [38]. Jonsson et al. [40] also state that the safety devices, road type, train traffic, and number of people living near a railway level crossing each have a significant impact on the probability of an accident.

Djordjević et al. [15] proposed a new approach to RLC security assessment using a non-radial data envelopment analysis (DEA) model. The proposed model is used to assess the effectiveness of railways in European countries in relation to the RLC safety level.

Magyari et al. [41] conducted a visibility study at railway level crossings by comparing point clouds from terrestrial laser scanning (TLS) and an unmanned aerial vehicle (UAV). It was found that the compared methods could produce contradicting results because the drone elevation model did not capture high and narrow columns, such as sign poles, and that the two survey methods could work effectively together.

Amaral et al. [21] studied laser-based obstacle detection at railway level crossings. They noted that detecting small obstacles (e.g., rocks ≈10 dm^3^) on an RLC is an open problem. They presented a system for obstacle detection on railway level crossings from 3D point clouds acquired with tilting 2D laser scanners. By allowing the detection of large and small obstacles (≥10 dm^3^) despite lighting conditions, they stated the system contributes to reducing the number of casualties in level crossings without reducing traffic flow.

This article concerns the safety status of railway level crossings in Poland. It contains the results of tests of the geometry of visibility triangles and assesses the impact on the safety of anomalies and non-compliance with regulations. The article draws attention to the need to implement measures that increase safety, with particular emphasis on surveying and diagnostic measurements to eliminate accidents and incidents on RLCs. The main objective of the research is to draw attention to a new approach to monitoring the geometry of visibility triangles of RLCs using an electronic total station and magnetic measuring square (MMS). The visibility is demonstrated, taking into account the angles of intersection of the road axis with the track axis of the line, including additional attributes to help analyze and evaluate the overall visibility conditions. The object of measurement is a railway level crossing of category D (passive) situated on a continuous welded rail. A case study examines the single-track railway line no. 144 Tarnowskie Góry—Opole Główne at 7.581 km, track no. 1, of the Silesian Voivodship (in Polish: Śląskie), Poland, characterized by the individual number: 144 007 581. At this location, the railway line is single-track, on which two-way traffic takes place.

In this work, the author’s research also highlights several controversies regarding particular factors:

- The threshold values of the angle of intersection of the road axis with the track axis of the line are different in different countries;
- There are differences in the permissible width and depth values of RLC flangeways in different countries;
- There are no uniform rules for measuring the location of road signs;
- The angle of intersection varies according to the location of the RLC in each type of conventional railway;
- Lack of uniform regulations and technical standards for monitoring RLC geometry and pedestrian crossings within the European Union.

The presented methodology, which is based on the integration of surveying prisms with MMS, confirmed the validity of their correct placement on the rail and the determination of characteristic measurement points for monitoring the geometry of visibility triangles of RLCs in conjunction with additional attributes. The measurement solution is tailored and closely related to the elements of the specific construction of the railway infrastructure, achieving a balance of safety and accessibility.

The introduced methodology for monitoring the geometry of RLC visibility triangles significantly improves the supervision of rail and road transport.

## 2. Related Work

Moayedfar et al. [20] investigate the calculation of sight triangle dimensions and unobstructed areas at railway level crossings in Iran. They draw attention to three categories:

- Approach area: Drivers should recognize the existence of a crossing in front of them, look for other trains or signs, and make appropriate decisions;
- Irreversible area: The area in which drivers decide to stop according to train distance and initial speed. If a correct decision to stop is not made in time, an accident will occur;
- Hazard zone: The area in which a stopped or moving vehicle may have collision with a stopped or moving train.

Tey at al. [42] applied VISSIM (in German: Verkehr in Städten—SIMulationsmodell) traffic microsimulation modeling based on data from field video recording. In addition, Młyńczak and Celiński in [43] used measurements based on an eye tracking device and an accelerometer (smartphone) installed onboard a test vehicle to monitor drivers of vehicles transporting dangerous and sensitive goods in the area of railway level crossings. The measuring system made it possible to study the characteristics of the driver’s process of perception of the external environment in the area of railway level crossings. For comparison purposes, an analogy was made to the measurement of train drivers’ behavior in the area of railway level crossings due to interaction of both road and rail traffic flows.

Bureika et al. [16] suggest assessing the safety of railway level crossings as a binary logical regression. Solving this regression results in an equation which consists of several variables and expresses the dependence of traffic safety at level crossings on several quantitative indicators, i.e., it estimates how a binary variable *Y* (safety at level crossings) depends on one or several variables. Variable *Y* is called the dependent (regressand) variable, and variables *X*_1_, *X*_2_, *X*_3_ are called independent variables (regressors). In the case of research [16], the dependent variable is safety level at level crossing *Y* and the independent variables are safety assessment criteria at level crossings, *X*_1_–*X*_7_.

Khan et al. [44] state that safety at highway rail grade crossings (HRCs) continues to be a serious concern despite improved safety practices. The authors developed a binary logit regression model to predict accident likelihood at HRCs by incorporating various contributory factors in addition to population within five miles of crossings.

Salmon et al. [45] noted that resolving the tension between efficiency and safety at rail level crossings is a key line of further inquiry.

Gikas [46] referred to surveying techniques for checking basic parameters of railway track geometry. To apply a geodetic mini prism (surveying mini prism) with a stylus tip as a standard solution for the measuring point below the top surface of the rail head, a special iron part was used to set the mini prism. Klug et al. [47] used the Leica Geodetic Mini Prism GMP111 with a surveying pole tip as a standard solution, which was placed in a special rail clamp drilling hole.

Urbančič et al. [48] suggested an improved approach for the control measurements of a ski-flying hill run in a case study of Planica. They presented a specially designed platform containing three measuring prisms to control the position of pipes (a metal tube is located on each side of the track to guide the trolley that mills tracks into the ice).

Marjetič et al. [49] and Kregar et al. [50] presented an alternative approach to controlling measurements of crane rails by applying a special platform on which two precise surveying prisms were fixed.

**Figure 1:**
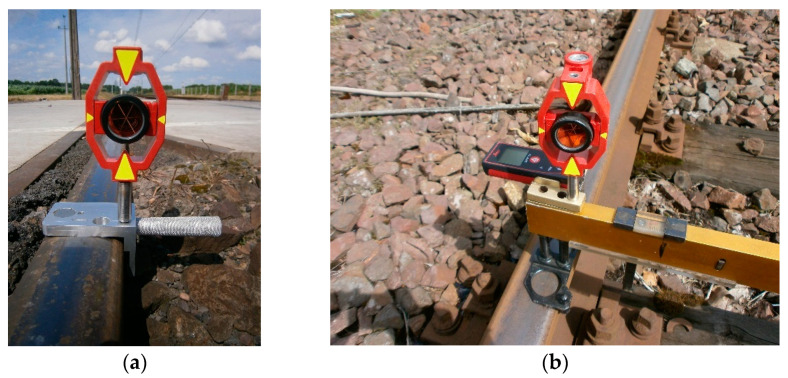
Integrated Geodetic Mini Prism Type 111 (GMP111) with: (**a**) magnetic measuring square (MMS); (**b**) gauge measure for measuring track and turnout parameters (GM) and laser distance meter (LDM).

This article is a continuation of previous research [2,3,51,52]. The works [2,3] include studies of the geometry of category D railway level crossings located on a double track line. In [2], the MMS device for monitoring the geometry of visibility triangles was used in measurements (Figure 1a), while in [3], a gauge measure (for measuring railway track and turnout parameters; GM) with adapters and Leica Geodetic Mini Prisms Type 111 (GMP111) and laser distance meter (LDM) (Figure 1b) were used.

The main vertical axis of the GMP111 mini prisms mounted on the GM coincides with the inner edge at a point 14 mm below the top running surface of the rail head (crown of the rail). The device is also compatible with other surveying prisms. The rear housing of the LDM mounted on the adapter coincides with the inner edge of the rail profile at a height of 14 mm below the top rolling surface of the rail head (Figure 1).

In [51], application of the magnetic measuring square in the measurement of the circular curve of rail transport tracks is presented, with particular reference to the measurement of versines and difference in rail length (rail shortenings). The study also focuses on the multifunctionality of its application, via integration with terrestrial laser scanning reference signals, a laser distance meter, surveying measuring disk (with a millimeter scale with concentric rings), and GMP111 [51,52].

Each measuring process assumes an object of measurement. However, each object must be localized in space; in general, there can be no space without objects [53]. Thus, regardless of the results and their mutual arrangement, it should be remembered that in the case of surveying measurements and continuous or periodic surveying monitoring, the choice of the measurement method and data processing method depends on the nature of the object and specific field conditions [54]. Dybeł [55,56] states that the nature of surveying work depends to a large extent on the type of the structure (construction) being studied and the accompanying phenomena. In addition, the interpretation of the acquired data is an important activity, and the possibility of taking cyclic measurements from the same points at a given time (in different time horizons) is highly important.

## 3. Methods and Measuring Instruments

To ensure the safety of RLCs located on a single-track railway line, it is necessary to monitor the geometry of visibility triangles. In this article, the geometry of visibility triangles of a railway level crossing of category D—passive is tested.

### 3.1. Geometry of Visibility Triangles RLCs—Passive

**Figure 2:**
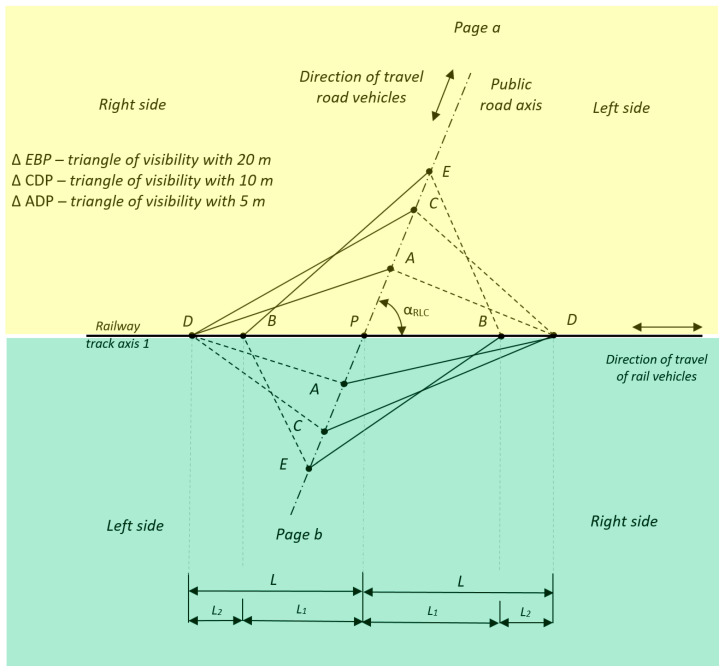
Surveying of visibility triangles.

The geometry of railway level crossings has a significant impact on the safety of railway transport [57]. RLCs and pedestrian crossings are used in a way that ensures railway and road traffic safety [1]. Measurement of the geometry of visibility triangles depends on regulations, among other factors [1,58]. Surveying of visibility triangles in RLCs requires the determination of points on the axis of the public road at distances of 4, 5, 10, 20, 30, and 34 m measured from the extreme rail for both of the pages “a” and “b” of the RLC (Figure 2). Next, the length of sections *L* and *L*_1_ and additional measurement points are determined (e.g., the width of the railway level crossing, location of road sign B-20 “stop”, G-3 “St. Andrew’s cross”, height of contact wires’ catenary, etc.).

Geometric conditions for the visibility of the train front from a public road require measurement (Figure 2):

1. From point *E*, i.e., 20 m from the RLC, the train’s front end shall be visible from point *B*, measured from the extreme rail along the road axis.
2. As the road vehicle approaches the railway level crossing, the train’s section of visibility should increase so that from 10 m (point *C*), the train’s front is visible at least from point *D*.
3. Due to local conditions, the front of the train shall be visible from a public road, at least 5 m, i.e., point *A* over the whole section *L*, starting from point *D*.
4. If the visibility is only 5 m, the B-20 “stop” road sign should be located on both sides of the railway level crossing.
5. Values of the angle of intersection of the public road axis with the track axis, α_RLC_, shall not be less than 60°, and the road sign G-3 “St Andrew’s cross in front of railway level crossing single track” shall be located.
6. Conditions of visibility of railway level crossings and pedestrian crossing should be assessed:
  - once per year, after the vegetation growth period, between June and September;
  - after every accident.
7. Visibility of a train from a public road for both pages of a railway level crossing, i.e., pages “a” and “b”.
8. Lengths of the train face visible from the public road *L* and *L*_1_ with one track. Length of *L* and *L*_1_ section is calculated from Equations (1)–(3):
  (1) $$L=5.5\cdot V_{max}$$
  (2) $$L_{1}=3.6\cdot V_{max}$$
  (3) $$L_{2}=L-\;L_{1}$$
  where *V_max_* is the highest permitted train speed in the RLC area in km/h.
9. Contact wire heights of the catenary.
10. The length of the railway level crossing. The length of RLC is a section of road whose end points are determined by the distance of 4 m from each of the extreme rails.
11. A section of road with a length of 30 m measured on the axis of the road on each page of the “a” and “b” railway level crossing from the end points, which is the so-called “access road”.
12. The width of the RLC, which is the width of the road crown at the railway level crossing.

### 3.2. Measuring Instruments: MMS and Electronic Total Station

To ensure the safe exploitation of railway level crossings, it is necessary to check the actual state of the geometry of their triangles of visibility. These tests are based on the measurement of the geometry of visibility triangles of RLCs of category D using an innovative magnetic measuring square and electronic total station, optimizing the conduct of surveying and diagnostic works and improving their quality. The surveying method for determining the geometry of visibility triangles is based on the classical surveying polar method, using an electronic total station and MMS with additional accessories for measuring point coordinates. The characteristic points located on the rail were determined by measuring the surveying position of the Leica GMP111 mini prism integrated from the MMS as target points. The electronic total station was positioned in the vicinity of the RLC measurement site, outside of the railway and road infrastructure. Measurements were taken from one observation station point (observation stand), ensuring a common coordinate system and uniform accuracy. The electronic total station was set in a stable position, allowing visibility of all desired measurement points of the railway track and the road axis. Significant points of measurement for the geometry of the RLC located on the track were signaled by the Leica GMP 111 surveying mini prism compatible and integrated with the MMS. Horizontal, vertical, and distance directions were measured. The prism constant is +17.5 mm (Leica Geosystems, Heerbrugg, Switzerland) [59,60]. The installation of the Leica GMP111 with the MMS ensured that the mini prism was stabilized directly above the measuring points. Its identification depends on subjective surveying evaluation.

The existing application of a standard surveying prism solution with survey pole, and the surveying pole tip to the transverse profile of the rail head, causes problems in determining the correct measuring point. The correct measuring point on the transverse profile of the rail head is available at a height of 14 mm below the top rolling surface of the rail head. Due to the specific shape of steel elements present in railway tracks, no scientific and technical publications were found that investigated control measurements of the geometry of visibility triangles using geodetic methods and techniques. The angular values of geometric conditions of the visibility of the train face from the road and the angles of road/railway track intersection are demonstrated with an accuracy to 1°.

**Figure 3:**
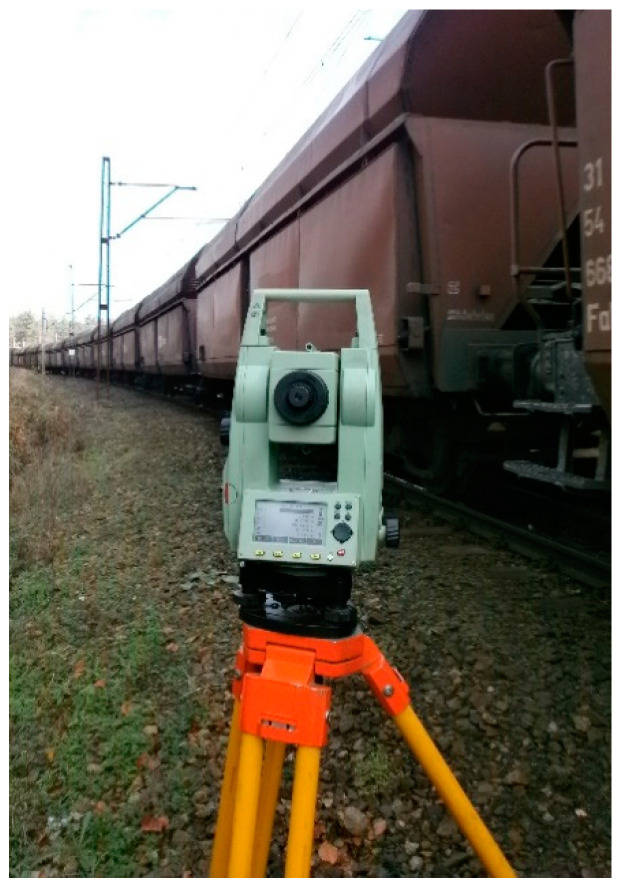
The electronic total station TC-407—measuring station.

The measurement was performed with the Leica TC407 electronic total station (Figure 3).

The accuracy standard deviation (H_sd_—horizontal direction, V_sd_—vertical angle/zenith angle (acc. to ISO 17123-3)) is 7″ (20^cc^) (Table 1) [60]. The operating temperature range is −20 to +50 °C. The electronic distance measurement (EDM) measuring program IR_Dokł/IR_Fine has a standard deviation (acc. to ISO 17123-4) of 2 mm + 2 ppm (Table 1) [60]. The accuracy of measured points m_p(pom)_ = 0.002 m, line-of-sight error (H_z_-collimation) = 0.0000 ^g^, and V_z_ index (vertical index error) = 0.0000 ^g^. Field work was carried out in favorable weather conditions in summer after the period of vegetation growth, taking into account atmospheric corrections (i.e., meteorological corrections).

**Figure 4:**
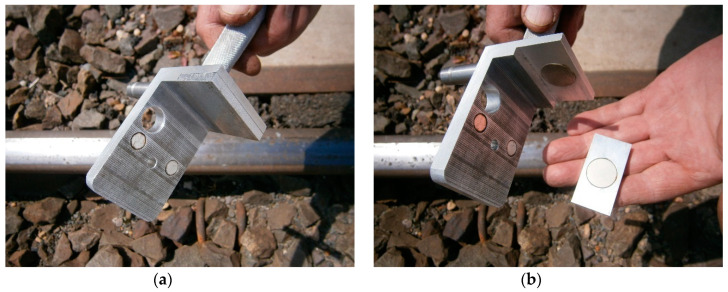
Magnetic base in MMS construction for mounting on metal parts: (**a**) with a reduction plate; and, (**b**) without a reduction plate.

The MMS was mounted on the external top rolling surface of the rail head type UIC60. On the upper surface of the MMS, a circular level (level bubble circular) was built up to ensure levelness. This consisted of a magnetic base fixed to the metal parts of the rail transport infrastructure (Figure 4), using neodymium magnets, which are an integral part of the MMS. Then, a universal connecting element with surveying prisms or prism adapters was used.

**Figure 5:**
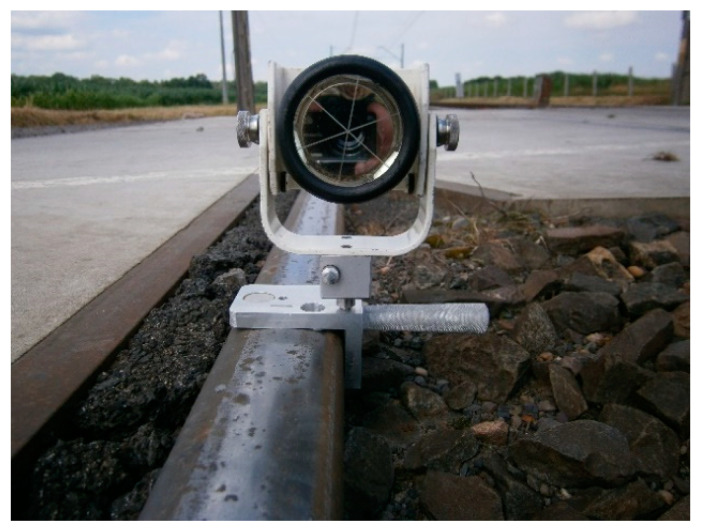
Geodetic standard prism (round) integrated with MMS.

The MMS provides for the installation of surveying prisms in relation to the lower edge of the rail head (or any other flat structural elements) by fitting a reduction plate (Figure 4a and Figure 5) or at a point 14 mm below the top running surface of the rail head, without a reduction plate (Figure 1a and Figure 4b). This allows fitting to the transverse profile of the rail head while also providing a reference point to the center of the surveying prism.

The MMS is also compatible with other prisms, depending on the required measurement needs.

## 4. Results and Discussion

### 4.1. Safety Status of Railway Level Crossings

RLCs are locations at which there is interaction between two modes of transport: rail and road. If safety is not appropriately maintained, the consequences of accidents at RLCs can be serious injuries and fatalities, in addition to significant material damage and restrictions, or disruptions to rail and road traffic.

On the active lines of the Polish railway network managed by 13 infrastructure managers (as of 31 December 2018), there were 12,801 railway level crossings and pedestrian crossings [61]. The number of RLCs and pedestrian crossings at individual infrastructure managers (administrator, IM) is shown in Table 2. The groups are as follows:

- RLCs of category D—6580 (51.4%);
- RLCs of category A—2415 (18.9%);
- RLCs of category C—1431 (11.2%);
- RLCs of category B—1270 (9.9%);
- RLCs of category F—616 (4.8%);
- Category E pedestrian crossings—489 (3.8%).

The factors that affect the exploitation of RLCs and thus the safety of traffic at single-level intersections of rail- and roadways are:

- Non-compliance with road traffic regulations by road users;
- Insufficient visibility of oncoming trains;
- Lack of correct geometry of visibility triangles;
- Lack of confidence in the technical performance of safety devices, e.g., long closing time; as a result, risky attempts are made to bypass the safety devices;
- Incomplete and illegible RLC marking;
- Too early activation or deactivation of safety devices.

**Figure 6:**
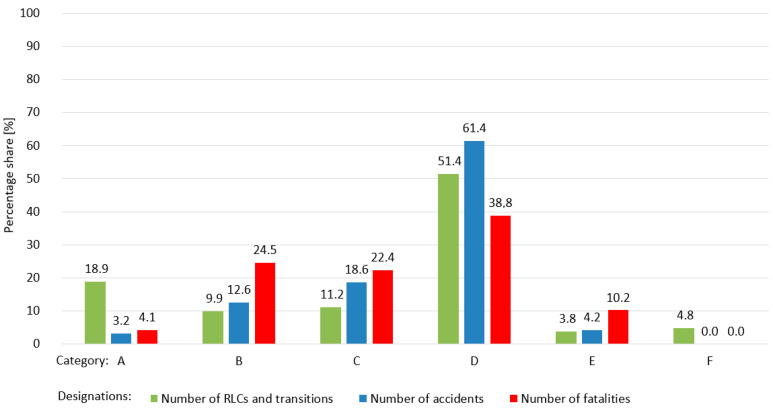
Percentage share of the number of railway level crossings and transitions, accidents, and fatalities, particularly RLC categories and transitions, in the Polish railway network.

A graphical interpretation of the number of railway level crossings and pedestrian crossings in individual categories is presented in Figure 6. The largest percentage (51.4%) are railway level crossings of category D.

RLCs are the point of contact between the railway and road systems [61]. A breakdown of RLCs on which accidents have occurred is presented in Table 3.

The percentage share of accidents on RLCs and transitions with railway tracks by category in 2018 is shown in Figure 6, which shows that the highest number of RLCs is in category D (61.4%). The distribution of fatalities is presented in Table 4.

In 2018, 49 people were killed at railway level crossings and pedestrian crossings. The percentage share of fatalities by RLC category is shown in Figure 6. The most dangerous rail level crossing is RLC category D, accounting for 38.8% of fatalities. The largest number of accidents and fatalities occur at category D railway level crossings on the Polish railway network, as these are not equipped with traffic protection systems and devices. This requires proper monitoring of the geometry of visibility triangles.

RLCs managed by PKP PLK S.A. (Polish State Railways Polish Railway Lines Joint Stock Company, in Polish: Polskie Koleje Państwowe Polskie Linie Kolejowe Spółka Akcyjna) are equipped with at least two yellow stickers with individual RLC identification numbers and emergency phone numbers. The yellow sticker contains three basic pieces of information:

- Individual identification number of the railway level crossing;
- Emergency number 112;
- The “emergency” numbers.

Reflective stickers are placed on the inner side of St. Andrew’s crosses if the crossing is a pedestrian crossing (cat. E), and the RLC is secured with signs (cat. D) or signs and traffic signals (cat. C). The size of the sticker is 40 × 11 cm. On railway level crossings with barriers (cat. B and cat. A), the stickers are located on the barrier drives (posts to which the barrier arm is attached). The size of the sticker is 5 × 20 cm.

In the event of a breakdown or other incident on an RLC, notification of the emergency number allows precise identification of the location of the incident using the individual identification number from the yellow sticker. Entering an individual railway level crossing identification number into a map geoportal or into the interactive railway map system directly displays the RLC on the map base with the RLC’s additional attributes.

**Figure 7:**
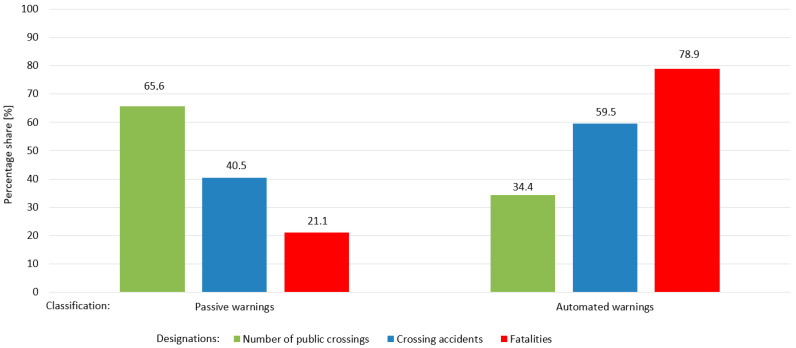
Percentage shares of number of railway level crossings (public crossings), accidents, and fatalities on the Canadian rail network.

Figure 7 shows the safety status of level crossings in the Canadian rail network. The Transportation Safety Board of Canada (TSB) gathers and uses data during the course of its investigations to analyze safety deficiencies and identify risks in the Canadian transportation system, with reference to crossing accidents by type and level of crossing protection [62]. The statistics presented reflect the TSB database as of 13 February 2018 for the reference year of 2017. The percentage shares of number of public crossings, crossing accidents, and fatalities on the Canadian rail network are shown in Figure 7, further classified by passive and automated warnings. In 2017, the number of public crossings was 16,524 (100%):

- passive warnings 10833 (65.6%);
- automated warnings 5691 (34.4%).

Number of crossing accidents was 116 at public crossings:

- passive warnings 47 (40.5%);
- automated warnings 69 (59.5%).

In turn, there were 21 crossing accidents at private crossings and five at farm crossings.

The number of fatalities was 19:

- passive warnings 4 (21.1%);
- automated warnings 15 (78.9%);

This is a worrying trend, particularly for automated warnings, and contrasts with the situation in Poland.

### 4.2. Case Study and Field Measurements

#### 4.2.1. Characteristics of the Research Object

**Figure 8:**
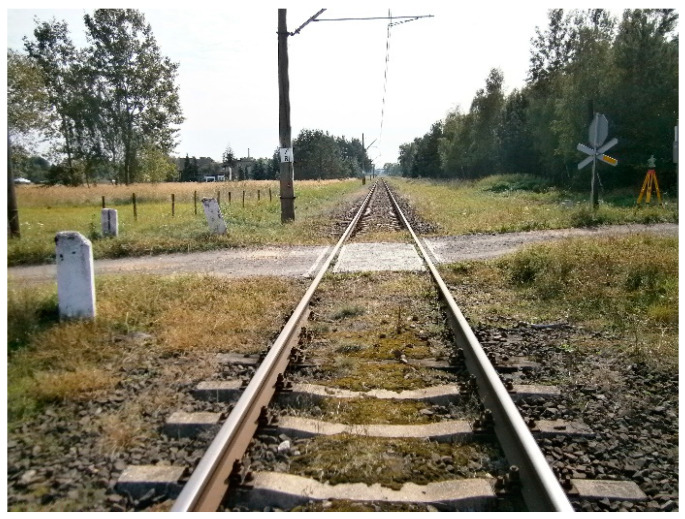
Railway level crossing no. 144 007 581 on a single-track railway line.

The measurement of the geometry of the visibility triangles took place on a category D—passive railway level crossing, located on a continuous welded rail. The location is on line no. 144 Tarnowskie Góry—Opole Główne at 7.581 km, in railway track no. 1, Silesian Voivodship, Poland, which is a railway line of the first category (1) in a built-up area. The individual identification number of the RLC is 144 007 581. The railway line is single-track, on which two-way traffic takes place (Figure 8).

The RLC constitutes a crossroads of the railway line no. 144 with a road on one level (public road), characterized by a ground surface. The dirt road on the access road to the RLC has a hard surface. The railway line is electrified, contact wires of the catenary network are located at a height of 5.48 m, and the track gauge is 1435 mm. The surface of the rail level crossing is equipped with concrete crossing slabs (level crossing slabs), which cover the RLC. The driving layer is laid inside and outside the railway track no. 1, with angle edge reinforcements (profiled edge reinforcements of the crossing slabs) at the whole height made of steel sheet, ensuring the passage of vehicles (motor vehicle) and railway rolling stock. A railway surface (permanent way) constitutes a structural unit consisting of UIC60 rails, concrete sleepers, ballast, and K-type rail fastenings of UIC60 rails for concrete sleepers, designed to carry the loads of rail vehicles on the subgrade. The railway surface within the RLC is of the same construction standard as the railway track adjacent to the RLC. The crown of the rails is arranged on one level, and the railway level crossing is located on a straight section of track. The maximum speed of passenger and freight trains is *V_max_* = 70 km/h.

#### 4.2.2. Results of the Methodology Used

In accordance with requirements [1], at a railway level crossing at which the contact wires catenary is suspended at a height of less than 5.6 m, an information board indicating the wires’ suspension height should also be placed. In the case of the RLC where measurements were carried out, such a board was placed on both sides of the RLC, because this height was 5.48 m. Table 5 contains the results of geometric values of linear triangles of visibility of the tested RLC no. 144 007 581.

Table 6 shows the results of the geometric angular values of the existing conditions of visibility of the train face from the road in relation to railway track 1 (according to the direction of travel of the road vehicle: left side, right side). These are defined for measurement points *A*, *C*, and *E*, respectively, located at 5, 10, and 20 m, on both track sides “a” and “b” (Figure 2).

**Figure 9:**
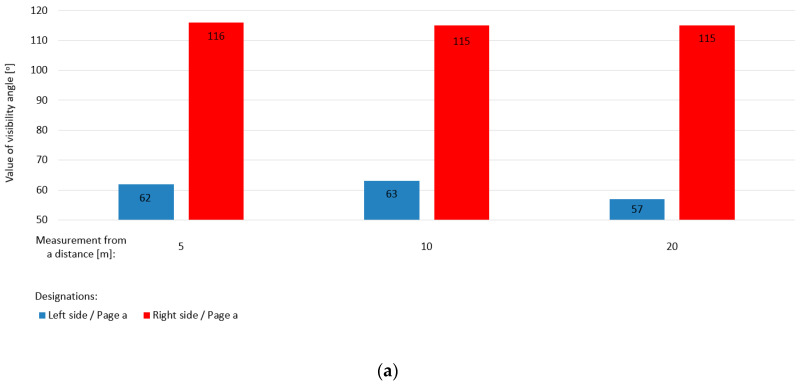

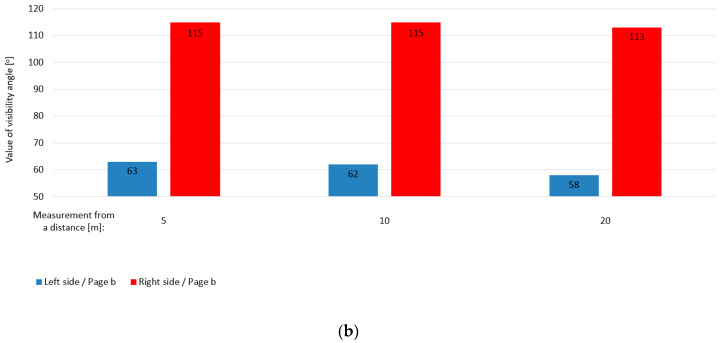
Values of the angles of visibility of the train front from the road for track no. 1: (**a**) left and right page “a”; (**b**) left and right page “b”.

The diagram in Figure 9 presents values of visibility angles from points *A*, *C*, and *E* for pages “a” and “b” of a railway level crossing. The visibility angles from the road for the left side are in the range 57–63° and for the right side are in the range 113–116°. The value of the intersection angle of the public road with the railway track axis, *α_RLC_*, is 99°; this value is not less than 60°. Thus, in accordance with [1], the road sign G-3 “St. Andrew’s cross before a single-track crossing” is placed.

In the RLC under examination, there is visibility only from point *A* (5 m), whereas from points *C* (10 m) and *E* (20 m), there are limitations on the visibility of the face of the train due to woodlands, bushes, tall trees, and the location of single family buildings with allotment gardens. The face of the train is visible from the public road from a distance of 5 m, i.e., point *A* over the whole section *L*, starting from point *D*, according to [1]. The visibility is maintained only from 5 m; thus, on the road on both sides of the RLC, the road sign B-20 “stop” is placed.

The value of the intersection angle *α_RLC_* is 99°; this is not less than 60° and hence the road signs G-3 “St Andrew’s cross in front of RLC single-track” and B-20 “stop” are placed at a distance of 5 m from the extreme rail.

If the distance of road sign G-3 “St. Andrew’s cross in front of RLC single track” from the extreme rail was more than 5 m, then the distance *L* should be increased by 0.25 *V_max_*, and *L*_1_ by 0.07 *V_max_*, for each meter of increased sign-setting distance [1]. If the intersection angle was less than 60°, then for every 5° below 60° for a distance of 20 m (section *EP*, Figure 2), when determining *L*_1_ from the acute angle side, it shall be increased by 1 m [1]. Research has shown that the current legal regulations do not specify rules for measuring the distance for setting the G-3 and B-20 road mark as:

- Perpendicular from the extreme rail;
- Parallel to the road axis.

**Figure 10:**
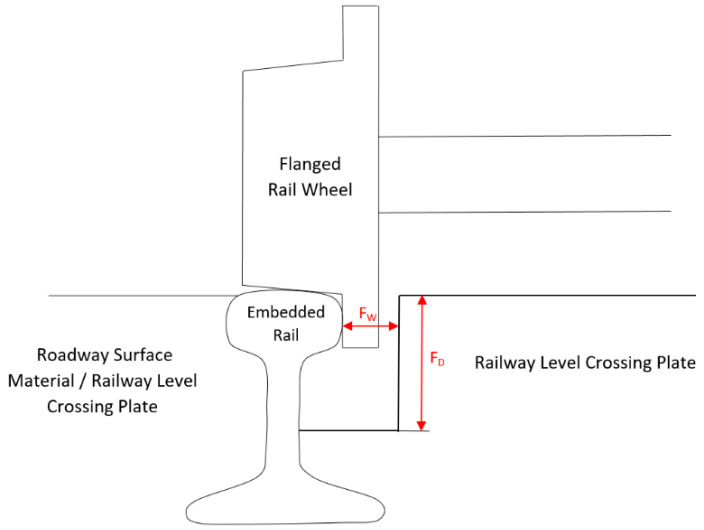
Width (*F_W_*) and depth (*F_D_*) of the flangeway of the RLC.

The studies were carried out by measuring the state of the flangeway width (*F_W_*) and flangeway depth (*F_D_*) on the RLC. Flangeways on railway level crossings should allow free passage of rail vehicle wheel flanges between the road surface laid inside the track and rails by providing a space between the running rail on the RLC and the edge of the road surface (Figure 10).

The depth of the flangeway on the RLC shall not be less than 38 mm and its width in straight track shall be at least 67 mm [2,3,58]. The measurement of the flangeway width was made 14 mm below the top rolling surface of the rail head using the MMS. The depth of the flangeway was measured from the top rolling surface of the rail head (Figure 10). Table 7 shows the values of the existing width and depth of the flangeway of railway track no. 1 at the railway level crossing no. 144 007 581. The measurement was carried out on the left edge, right edge, and RLC axis, both for the left (*R_L_*) and right (*R_R_*) rail (in relation to the kilometer in the railway line). Inadmissible values are marked in red.

#### 4.2.3. Discussion

Nedeliaková et al. [8] correctly state that the relevant facts are specific to individual railway level crossings under certain conditions, and each RLC thus needs to be monitored separately. In [8], it is noted that a wider debate is needed on issues such as the risk of accidents and incidents at railway level crossings. In [11], it was found that in the Czech Republic, the majority of accidents happen on RLCs only with flashing lights. The results confirmed the influence of the traffic volume on safety, in addition to speeding and individual viewing distances. It was noted that this dependence may change in line with the distance from the RLC. Although approaching the viewing distance was associated with a decrease in the frequency of accidents, an inverse trend in stopping at the viewing distance was found. L’upták et al. [63] drew attention to basic deficiencies in the field of railway crossing safety. They state that it “is the willingness, or lack thereof, of the European Commission and the government to resolve the issues concerning the maintenance of level crossings” and “it is clear that the safest level crossing is one that does not exist”. Single-level RLCs have long existed and will continue to exist for a very long time and thus require appropriate surveying and diagnostic monitoring. The trend of most accidents occurring at RLCs with flashing lights in the Czech Republic is also confirmed by the results on RLCs of the Canadian railway network.

Risk homeostasis and behavioral adaptation—once subjective safety is increased by providing better visibility—may cause drivers to become more reckless and pay less attention.

Rail infrastructure managers are increasingly limiting the number of RLCs. However, there has been an increase in the number of road vehicles. The challenge for infrastructure managers (road and rail) is to take action with a large number of road vehicles and an increasing number of trains. Reducing the number of RLCs is a difficult task. Most RLCs are required because users of road vehicles have the right to cross the tracks due to local conditions and road categories. This also applies to the issue of communities and their behavior, particularly local communities, who do not wish to lose local railway level crossings with safe conditions for their use. Moayedfar et al. [20] note that safety is the most important factor affecting the quality of transport, both for workers and users of transport infrastructure. RLCs present a safety issue that is increasingly drawing the attention of the public and infrastructure managers.

Tey et al. [42] recognize that security on RLCs is increasingly attracting public attention. Salmon et al. [45] emphasize that work domain analysis (WDA) clearly shows that railway level crossing systems are complex environments with many interacting elements (human and non-human), many competing goals, many values and priorities, and many failure paths. Social organization and co-operation analysis (SOCA) shows the division of tasks and functions between people, technologies, and objects. The analysis shows that it is potentially possible to develop a more appropriate division of tasks and functions in the environment of RLCs [45].

Kobaszyńska-Twardowska et al. [17] state that despite the growing role of robotics, automation, and other integrated technological processes, human operators remain the most important element in both object and process control systems. Despite the introduction of various technical devices supporting the driver, driver effectiveness and safety have the greatest influence on system operation.

Improving safety at passive railway level crossings is an ongoing global problem. At these RLCs, there are no active warning systems supporting drivers’ decision making. These crossings depend on road users noticing approaching trains when making a decision to enter the RLC [17]. It is highly important to carry out surveying and diagnostic monitoring of railway level crossings, especially with regard to the geometry of visibility triangles. Młyńczak and Celiński [43] emphasize that RLC issues concern the construction of appropriate approaches (access roads) at railway level crossings. In addition, often, visibility is limited by existing buildings in the area of railway level crossings. The geometry of RLC limitations in terms of maintenance is also influenced by sleepers that are not supported or suspended at a railway level crossing, which need to be raised [37].

The rules for measuring the geometry of visibility triangles of a single-track line are characterized by other dependencies, such as the length of the sections of visibility of the face of a train from a public road, *L* and *L*_1_. In the case of a multiple-track line, these sections require that the state of the intertrack space is taken into account.

The geometry of a railway level crossing relates to the central symmetry with respect to the fixed point *P*, called the center of symmetry (Figure 2). The proposed approach allows for simultaneous determination of the geometry of visibility triangles using the electronic total station from a single measuring station with MMS. It is based on the polar measurement method. The proposed approach is an improvement compared to previous methods because it allows measurement of the geometry of visibility triangles faster and more accurately.

The research also showed the following discrepancies:

1. Differences in the angle of intersection of the road axis with the railway track axis. Threshold values of this angle differ in different countries. Studies have found the following variations:
  - For projected railway level crossings and pedestrian crossings on standard and broad-gauge railway lines, Poland is allowed to use an intersection angle meeting the condition 120° ≥ *α* ≥ 60°. However, the standard crossing angle should be 90° [1];
  - In Canada, for all new grade crossings and changes made at existing grade crossings, a grade crossing angle, measured from the tangent of the centerline of the road approach at the crossing surface to the tangent of the centerline of the line of railway, shall, where the railway design speed is more than 25 km/h (15 mph), be [64]:
    − Not less than 70 and not greater than 110 degrees for grade crossings without a warning system; or
    − Not less than 30 and not greater than 150 degrees for grade crossings with a warning system.
  - Republic of Slovenia [65]:
    − The intersection of the road with the rail line at a RLC must be as close as possible to a right angle, and the angle must not be less than 75°;
    − Notwithstanding the point above, on a protected RLC, the crossing of the road with the railway line is exceptionally permissible at an angle of less than 75°, but not less than 45°, if very demanding conditions for road construction are given;
    − The road operator must prohibit these participants from driving over RLCs that are not structurally arranged in such a way as to ensure the safe crossing of bicycles, with traffic signals.
2. Differences in the permissible width and depth values for RLC flangeways in different countries.
3. There are no uniform rules for measuring the location of road signs as perpendicular to the nearest extreme rail or parallel to the road axis to the nearest extreme rail. The introduction of these principles is reflected in the length of the location of sections *L* and *L*_1_.
4. The angle of the intersection depending on the location of the RLC in a railway line: narrow-gauge, normal-gauge, wide-gauge, or at a railway siding.
5. Lack of uniform regulations and technical standards for monitoring RLCs’ geometry and pedestrian crossings within the European Union.

Gikas [46] referred to surveying techniques for checking basic parameters of railway track geometry. A special iron part was used to apply a surveying mini prism with a stylus tip as a standard solution to the measuring point below the top rolling surface of the rail head. This needs to be held in a vertical position. However, this method of applying the surveying mini prism to the rail head is controversial due to systematic errors and lack of stability.

The main disadvantage of using a Leica GMP111 mini prism with a pole and tip (or other surveying prism) as a single prism method applied to a point of measurement located on the transverse profile of the rail head is that it is problematic to determine, and position it at, the target point. Additionally, a problem exists with the repeatability of the selected measurement point. It is practically impossible to place the prism on the same point several times. The use of an integrated MMS with surveying prisms prevents such errors.

In the conducted research [41], which involved the integration of TLS and UAV measurement methods, it was found to be beneficial to link these methods with the new approach for the measurement of visibility triangle geometry using an electronic total station and MMS as a hybrid method. The application of this approach may be beneficial, allowing more complete information to be gathered that will lead to more accurate recognition of approaching emergencies.

## 5. Conclusions

It is necessary to conduct research to improve safety at railway level crossings and pedestrian crossings. However, the public is also obliged to comply with the applicable road user rules. Modern technology also contributes to improving safety at railway level crossings and pedestrian crossings, thus providing better protection.

This article examines the control measurements of the geometry of visibility triangles using an electronic total station and MMS with additional attributes. The research confirmed the usefulness of the presented measurement methodology, particularly in terms of the importance of geometry control. To achieve the highest level of efficiency, an optimal control measurement methodology should be applied. The described method simplifies the process of control measurements of the geometry of visibility triangles and ensures its accuracy.

The detail and impartiality of the specification of characteristic points is the main advantage of the proposed solution. The integrated Leica GMP111 surveying mini prism with MMS or gauge measure ensures that the point is correctly related to the transverse profile of the crown of the rail. The classic method used to date, which is based on the use of a surveying mini prism with a ranging pole and a surveying pole tip, combined with the polar method, suffers from the problems during measurement of repeatability and determination of the characteristic point. The use of the Leica GMP111 integrated surveying mini prism with MMS or with a GM as a physical stabilization of a characteristic measuring point ensures that it can be correctly determined, measured, and repeated at the same point. The proposed methodology prevents defects that occur in the use of geodetic prisms with a ranging pole and surveying pole tip in the range of characteristic points located on elements of the metal structures of railway infrastructure. The proposed methodology for control measurements can also be used to monitor the geometry of other infrastructures to ensure good quality, analysis, and evaluation.

In future work, I intend to analyze the state of permissible values of crossing angles contained in legal regulations in other countries and investigate the state of RLC geometry at intersections of railway lines with roads with more than two directions of travel.

## 6. Patents

Magnetic measuring parallel motion protractor and its application. Patent PL 235051 B1. (Zespół przekładnic magnetyczno—pomiarowych do pomiaru parametrów geometrycznych torów i rozjazdów. Patent PL 235051 B1).

Messages from the Patent Office RP, 05, (2020) Warsaw.

Wiadomości Urzędu Patentowego RP, 05, (2020) Warszawa.

## Figures and Tables

**Table 1:** Main technical characteristics of the Leica Geosystems TC407 total station.

Technical Data		Value
Accuracy standard deviation H_sd_, V_sd_ (acc. to ISO 17123-3)		7″ (20^cc^)
Compensator—setting accuracy		2″ (7^cc^)
Operating temperature		−20 °C to +50 °C
Accuracy standard deviation—electronic distance measurement (EDM) measuring Program (acc. to ISO 17123-4)	IR_Fine	2 mm + 2 ppm
	IR_Fast	5 mm + 2 ppm
	Tracking	5 mm + 2 ppm
	IR_Tape	5 mm + 2 ppm
Carrier wave		0.780 μm
EDM type	Coaxial	
Line-of-sight error (H_z_-collimation)		0.0000 ^g^
V_z_ index (vertical index error)		0.0000 ^g^

**Table 2:** Number of Railway level crossings (RLCs) and crossings of individual administrators.

L.p.	Infrastructure Manager	RLC Category and Pedestrian Crossing						Total
		A	B	C	D	E	F	
1	PKP Polskie Linie Kolejowe S.A.	2337	1255	1415	6200	468	584	12,259
2	PKP Linia Hutnicza Szerokotorowa Sp. z o. o.	37	5	7	184	9	16	258
3	PKP Szybka Kolej Miejska w Trójmieście Sp. z o. o.	0	0	0	1	1	0	2
4	CTL Maczki-Bór S.A.	6	0	0	11	0	2	19
5	Infra SILESIA S.A.	14	0	0	15	2	2	33
6	Jastrzębska Spółka Kolejowa Sp. z o. o.	8	1	1	17	2	2	31
7	Kopalnia Piasku Kotlarnia—Linie Kolejowa Sp. z o. o.	6	0	0	70	1	7	84
8	PMT Linie Kolejowa Sp. z o. o.	4	2	2	29	0	0	37
9	CARGOTOR Sp. z o. o.	0	0	0	0	0	0	0
10	Euroterminal Sławków Sp. z o. o.	3	0	0	4	0	0	7
11	Dolnośląska Służba Dróg i Kolei we Wrocławiu	0	3	2	20	1	0	26
12	Warszawska Kolej Dojazdowa Sp. z o. o.	0	4	4	29	5	3	45
13	Pomorska Kolej Metropolitalna S.A.	0	0	0	0	0	0	0
	Σ	2415	1270	1431	6580	489	616	12,801

**Table 3:** RLCs with accidents.

RLC Category	Number of Accidents	Percentage
D	132	61.4
C	40	18.6
B	27	12.6
A	7	3.2
E pedestrian crossings	9	4.2
F	0	0.0

**Table 4:** RLCs with fatalities.

RLC Category	Number of Fatalities	Percentage
D	19	38.8
B	12	24.5
C	11	22.4
E pedestrian crossings	5	10.2
A	2	4.1
F	0	0.0

**Table 5:** Geometric linear values of the triangles of visibility of the studied RLC: 144 007 581.

Geometric Values		Value	Units
Name	RLC Page		
Longitudinal gradient of the road on the access road to the railway track over a length of 30 m from the end points of the RLC	“a”	0.63	%
	“b”	0.78	%
RLC length	-	9.528	m
Crown width of the road on RLC	“a”	3.600	m
	“b”	3.400	m
Average roadway width on RLC	-	3.285	m
Average width of the roadway on access roads	“a”	3.102	m
	“b”	2.886	m
The length of a straight road section, measured from the extreme rail	“a”	33.910	m
	“b”	34.084	m
Length of visibility sections $L=5.5\cdot V_{max}$	-	385.000	m
Length of visibility sections $L_{1}=3.6\cdot V_{max}$	-	252.000	m
Length of visibility sections $L_{2}=L-\;L_{1}$	-	133.000	m

**Table 6:** Geometric angular values of existing conditions of train front visibility from the road for track 1.

Measurement of Visibility Conditions from the Road (m)											
5 (m)				10 (m)				20 (m)			
railway track side				railway track side				railway track side			
“a”		“b”		“a”		“b”		“a”		“b”	
(°)		(°)		(°)		(°)		(°)		(°)	
left	right	left	right	left	right	left	right	left	right	left	right
62	116	63	115	63	115	62	115	57	115	58	113

**Table 7:** Existing RLC flangeway values.

Railway Track Number	Rail	Width and Depth of the Flangeway of the RLC (mm)					
		Left Edge		RLC Axis		Right Edge	
		Width (*F_W_*)	Depth (*F_D_*)	Width (*F_W_*)	Depth (*F_D_*)	Width (*F_W_*)	Depth (*F_D_*)
1	*R_L_*	72	25	70	65	62	80
	*R_R_*	55	67	70	80	85	72

The red color indicates exceeded values. *R_L_*—Left rail; *R_R_*—Right rail.
